# Biphasic composite of calcium phosphate-based mesoporous silica as a novel bone drug delivery system

**DOI:** 10.1007/s13346-019-00686-3

**Published:** 2019-12-09

**Authors:** Magdalena Prokopowicz, Adrian Szewczyk, Adrianna Skwira, Rafał Sądej, Gavin Walker

**Affiliations:** 1 Faculty of Pharmacy, Department of Physical Chemistry, Medical University of Gdańsk, Hallera 107, 80-416, Gdansk, Poland; 2 Department of Molecular Enzymology, Intercollegiate Faculty of Biotechnology, University of Gdansk and Medical University of Gdansk, Gdansk, Poland; 3 Bernal Institute and Department of Chemical Science, University of Limerick, Limerick, Ireland

**Keywords:** Drug delivery, Mesoporous silica, Hydroxyapatite, Doxycycline

## Abstract

We reported the new biphasic composites of calcium phosphate and mesoporous silica material (CaP@MSi) in the form of powders and pellets as a potential bone drug delivery system for doxycycline hydrochloride (DOX). The CaP@MSi powders were synthesized by cationic surfactant-templating method. The effects of 10, 20, and 30% CaP content in the CaP@MSi powders on the molecular surface structure, the cytotoxicity against osteoblast cells *in vitro*, and the mineralization potential in simulated body fluid were investigated*.* The CaP@MSi characterized by the highest mineralization potential (30% CaP content) were used for DOX adsorption and pelletization process. The CaP which precipitated in the CaP@MSi composites was characterized as calcium-deficient with the Ca:P molar ratio between 1.0 and 1.2. The cytotoxicity assays demonstrated that the CaP content in MSi increases osteoblasts viability indicating the CaP@MSi (30% CaP content) as the most biocompatible. The combination of CaP and MSi was an effective strategy to improve the mineralization potential of parent material. Upon immersion in simulated body fluid, the CaP of composite converted into the bone-like apatite. The obtained pellets preserved the mineralization potential of CaP@MSi and provided the prolonged 5-day DOX release. The obtained biphasic CaP@MSi composites seem to have an application potential as bone-specific drug delivery system.

**Electronic supplementary material:**

The online version of this article (10.1007/s13346-019-00686-3) contains supplementary material, which is available to authorized users.

## Introduction

The infection of bone (*osteomyelitis*) belongs to diseases difficult to diagnose and treat. Chronic *osteomyelitis* caused by bacterial bone infections occurs mainly in adults, usually as a consequence of open bone injuries, bone reconstruction, or implant insertion. In clinical practice, the *osteomyelitis* is treated surgically with the simultaneous implementation of the parenteral antibiotic therapy [1]. The main problem of the antibiotic therapy is low bioavailability of drugs in bones and thus inadequate therapeutic concentration of the antibiotics in infected area [2]. Therefore, increased attention has been paid to local bone drug delivery systems with prolonged antibiotic release to maintain the drug concentration at suitable level in bone tissue for the entire period of treatment [3, 4]. Another promising feature of such bone drug delivery systems is mineralization potential connected with self-formation of biological calcium phosphate on their surface which supports the bone regeneration [5].

The combined effect of the high drug loading capacity of mesoporous silica materials (MSi) for antibiotic delivery together with mineralization potential of calcium phosphate minerals (CaP) is an outstanding perspective for bone therapy purposes. The CaP are widely used in medicine and dentistry because of their excellent biocompatibility and bioactive properties [6]. In the context of bone regeneration, CaP have been used in various forms, ranging from amorphous calcium phosphate to crystalline hydroxyapatite (Hap) [7].

The MSi have been recently suggested as candidates for bone drug carriers due to their mesoporous arrangement, specific surface area, chemical composition, and biocompatibility. However, the MSi materials have generally poor mineralization potential studied in simulated body fluid. The typical induction times for Hap nucleation on the surface of MSi are in the range of 30–60 days [8]. To improve mineralization potential of MSi, the osteogenic compounds were embedded into the silica network [9, 10]. After addition of osteogenic compounds, semi-crystalline carbonate apatite formation with composition and morphology similar to bone apatite was observed on silica surface. Unfortunately, the modification of MSi materials with osteogenic ions results in disruption of their ordered porous structure; consequently, reproducible and effective drug adsorption is limited. Therefore, it is believed that the combination of the two components of CaP and MSi might give a better result. This biphasic composite of calcium phosphate-based mesoporous silica material (CaP@MSi) can act as a bifunctional drug delivery system with mineralization potential and prolonged drug release. However, it is worth mentioning that the MSi materials obtained *via* templating method exist in the form of powders characterized by the relatively good adhesive properties and low bulk density what causes problems in their direct pharmaceutical usage. Moreover, the fast release of water-soluble drug in a burst stage has been frequently observed for drug-loaded MSi. One of the difficulties with burst release is its unpredictable nature. Additionally, the released amount of drug during burst stage is hard to be significantly controlled [11]. Nowadays, one of the most common strategies overcoming the aforementioned disadvantages is the synthesis of hierarchically structured materials in the nanoscale based on the MSi powders [12, 13]. However, the production of drug-loaded silica nanoparticles requires advanced skills and technologies to provide crucial factors such as the stability of the nanoparticles which tend to agglomerate; the suitable ratio of drug release to nanoparticles biodegradation; and the stimuli-controlled drug release, necessary for targeted drug delivery systems. Herein, we would like to propose a simple solution in the macroscale basing on the pharmaceutical technology. We used the pelletization technique to prepare spherical granules composed of the CaP@MSi powders. The applied pelletization process is based on wet granulation, extrusion, and spheronization steps in which the fine powders and excipients are finally converted into small, spherical units, which are referred to as pellets. The form of pellets was chosen due to its desirable properties such as improved mechanical resistance, great flow properties, low susceptibility to dose dumping, and regular shape suitable for further pharmaceutical processing.

The idea of our studies is to design, synthesize, and evaluate the application potential of CaP@MSi composite materials in the form of spherical granules—pellets as implantable, bifunctional bone drug delivery system. After surgical implantation, such system might provide the prolonged drug release directly in the infected bone tissue and support the bone regeneration *via* formation of surface hydroxyapatite with morphology and composition similar to human bone apatite.

Consequently, the first aim of the present paper was to synthesize a new biphasic composite composed of mesoporous silica materials (MSi) and calcium phosphate (CaP) using a cationic surfactant as the structure-directing agent. The cationic surfactant, cetyltrimethylammonium bromide (CTAB), is one of the most studied in the synthesis of both the MSi and CaP materials. It forms spherical micelles with ∼ 2–3-nm diameter when critical micelle concentration is exceeded (∼ 1 mM in water at room temperature). The formed micelles undergo sphere-to-rod transition above ∼ 250 mM [14]. The biphasic composite of MSi@CaP was further formed in the wet precipitation process onto the self-assembling rod-like micelles of CTAB. After the synthesis of biphasic composites, we described the Ca:P molar ratio of the CaP found in MSi as a function of both the CaP precursors amount in synthesis and the immersion time in simulated body fluid. Next, we studied the toxicity against osteoblast cells. Finally, we prepared spherical granules (pellets) based on the optimal formulation of biphasic composite of CaP@MSi with adsorbed drug and excipients. Doxycycline hydrochloride (DOX) was chosen as a model drug. It is highly water soluble antibiotic widely used in the pharmacological treatment of *osteomyelitis* [15]. The mineralization potential of the pellets and the DOX release studies were investigated to show their potential in two biomedical applications: bone tissue regeneration and local drug delivery.

## Materials and methods

### In situ synthesis of the composite CaP@MSi

The parent MSi powders (100MSi) were synthesized using a surfactant templating method with tetraethyl orthosilicate (TEOS, 98%, Gelest) as a silica oxide source and cationic surfactant N-cetyltrimethylammonium bromide (CTAB, CH_3_(CH_2_)_15_N(CH_3_)_3_Br, 99.0%, Sigma-Aldrich) as a template. The pure CaP (100CaP) were synthesized by following the same templating method as for the 100MSi, where anhydrous calcium chloride (CaCl_2_, 99.0%, Sigma-Aldrich) was used as a calcium source and anhydrous di-potassium hydrogen phosphate (K_2_HPO_4_, 99%, Sigma-Aldrich) as a phosphorous source and CTAB as a template. The amounts of CaCl_2_ and K_2_HPO_4_ were adopted to the chemical reaction (Eq. 1), following the wet synthesis of Hap in the presence of ammonia [16]:

1 $$ 10{\mathrm{Ca}\mathrm{Cl}}_2+{6\mathrm{K}}_2{\mathrm{HPO}}_4+{2\mathrm{H}}_2\mathrm{O}\to {\mathrm{Ca}}_{10}{\left({\mathrm{PO}}_4\right)}_6{\left(\mathrm{OH}\right)}_2+12\mathrm{KCl}+8\mathrm{HCl} $$

The composites of MSi with CaP (CaP@MSi) were obtained by combining together the MSi and CaP precursors in different proportions: amounts of precursors in the synthesis of CaP@MSi are shown in Table 1. In brief, 125 g of water, 12.5 g of ethanol (> 99%, Sigma-Aldrich), 9.18 g of aqueous ammonia (25 wt% in water, Sigma-Aldrich), and 2.39 g of template (CTAB) were stirred together in polypropylene beaker on magnetic stirrer (300 rpm) for approx. 15 min at room temperature until the homogenous solution appeared. The pH of the obtained solution was 10. Then, the relevant amounts of K_2_HPO_4_, CaCl_2_, and TEOS (Table 1) were added and the resulting mixture was continuously stirred for 2 h. Next, the hydrothermal treatment of the mixture was carried out at 90 °C for 5 days without stirring. The resulting solid product was recovered by vacuum filtration, washed with 100 ml of absolute ethanol, and dried at 40 °C for 1 h. The CTAB template was removed from the solid product using calcination in air for a period of 6 h at 550 °C (heating rate of 1 °C/min) in a muffle furnace (M-525, II series). The final CaP@MSi samples in the form of powders were micronized in grinder (Mortar Grinder Pulverisette 2, Fritsch) for 10 min at 50 rpm. The 200–500-μm fraction was used for further studies. The final compositions have been named as 100%MSi, 10%CaP@MSi, 20%CaP@MSi, 30%CaP@MSi, and 100%CaP taking into the account the theoretical amount of Ca and P precursors used in the synthesis (Table 1).

**Table 1:** Nominal compositions (wt%) and amounts of reactants (g) of the calcium phosphate/mesoporous silica composites

Composition (wt%) (system name)	TEOS	CaCl_2_	K_2_HPO_4_
100%MSi (100MSi)	10.03	0	0
10%CaP90%SiO_2_ (10CaP@MSi)	9.03	0.334	0.314
20%CaP80%SiO_2_ (20CaP@MSi)	8.02	0.668	0.628
30%CaP70%SiO_2_ (30CaP@MSi)	7.02	1.002	0.942
100%CaP (100CaP)	0	4.612	4.336

### Characterization of CaP@MSi

The molecular structure of as-synthesized CaP@MSi samples was characterized using a Fourier transform infrared spectroscopy (FTIR, Jasco model 410, 4 cm^−1^ resolution), using the potassium bromide (KBr) disk technique. The parent 100MSi, 100CaP, and commercial crystalline hydroxyapatite (Hap; Ca_10_(PO_4_)_6_(OH)_2_) powder (> 98%, Sigma-Aldrich) were also studied for the comparative purposes. For better comparison of synthesized materials, the FTIR spectra were normalized to maximum absorption of dominant peak at ∼ 1080 cm^−1^ attributed to the asymmetric stretching of siloxane bands. The crystalline phases in the CaP@MSi were characterized by the wide-angle powder X-ray diffraction (PXRD). PXRD analyses were taken with an Empyrean/PANalytical XRPD diffractometer using CuKα1 radiation, operating at 20 kV and 40 mA. The samples were scanned from 10 to 60 in 2θ with a scan speed of 2.0°/min. Joint Committee on Powder Diffraction Standards (JCPDS) files were used to compare the positions of diffracted planes.

The morphology and particle size of synthesized materials were measured by using the scanning electron microscopy (SEM). The Ca:P molar ratio values were determined according to the data collected from different parts of the samples using the energy dispersive X-ray spectroscopy (EDX). SEM-EDX measurements were obtained on a Quanta 3D FEG and Hitachi SU-70 electron microscopes at an acceleration voltage in the range of 3–30 kV. Samples for SEM-EDX were fixed on carbon tape and coated with gold for 1 min. Transmission electron microscopy (TEM) images were obtained using a Tecnai G2 T20 X-TWIN electron microscope at an accelerating voltage of 200 kV. The samples were dispersed in hexane over copper grids and dried in the air.

The porosity of the synthesized materials was determined using nitrogen sorption experiments. The adsorption/desorption isotherms of nitrogen at 77 K were measured with an automated apparatus ASAP 2020 (Micromeritics, USA). Prior to the measurements, the samples were degassed for 2 h at 160 °C. The specific surface area was calculated with a multipoint Brunauer–Emmett–Teller (BET) method. Total pore volume was derived from the amount of adsorbed nitrogen at a relative pressure of p/p_o_ = 0.99. The pore size distribution was determined from the adsorption branch of the isotherm by using the Barrett–Joyner–Halenda (BJH) method.

### In vitro mineralization assay of CaP@MSi

The mineralization potential of the CaP@MSi composites (200–500-μm particles size) was investigated using simulated body fluid proposed by Kokubo and Takadama [17]. To prepare 1 L of SBF, 7.996 g of NaCl, 0.350 g of NaHCO_3_, 0.224 g of KCl, 0.228 g of K_2_HPO_4_·3H_2_O, and 0.305 g of MgCl_2_·6H_2_O were dissolved in 750 mL of deionized water. Then, 39.0 mL of hydrochloric acid (1 M) was added to the solution. Next, 0.278 g of CaCl_2_, 0.071 g of Na_2_SO_4_, and 6.057 g of TRIS were sequentially dissolved in the solution. Finally, the solution was adjusted to pH = 7.4 with 1 M hydrochloric acid and diluted to 1 L using purified water. SBF precursors were used as received from Sigma-Aldrich.

In order to investigate the mineralization potential of the CaP@MSi composites, samples were immersed for 28 days in simulated body fluids (SBF) using 1 mg of composite per 1 mL of SBF. Briefly, 100 mg of composite was immersed in 100 mL of SBF in polypropylene forms. The experiment was performed in water bath (Witeg WSB-30) at 37.0 °C under the stirring conditions (70 rpm). After every 24 h, the samples were centrifuged, the supernatant was collected using pipette, and the fresh portion of the SBF was poured [18]. After each 7 days of mineralization assay, the samples were filtered, dried at 40 °C, weighted and investigated by using SEM-EDX method in order to determine the Ca:P molar ratio and the morphological structure of the samples. Next, the whole procedure was repeated providing 1 mg:1 mL ratio between materials and SBF.

### In vitro cytotoxicity assay of CaP@MSi

Human fetal osteoblastic cell line (hFOB 1.19) was obtained from American Type Culture Collection (cat. no. CRL-11372™). Cells were cultured in 1:1 mixture of Ham/s F12 Medium Dulbecco’s Modified Eagle’s Medium, with 2.5 mM L-glutamine (without phenol red) (Sigma-Aldrich), 15 mM HEPES, and sodium bicarbonate, supplemented with 10% fetal bovine serum and penicillin/streptomycin (100 U/mL/100 μg/mL) at 34 °C in a humidified atmosphere of 5% CO_2_. Medium was replaced every 2–3 days. Cells were passaged for a maximum of 3–4 months post resuscitation and regularly tested for mycoplasma contamination by two methods: DNA staining with 4,6-diamidino-2-phenylindoledihydrochloride (DAPI) and MycoAlert Mycoplasma Detection Kit (Lonza, Basel, Switzerland). The influence of parent 100MSi, 10CaP@MSi, 20CaP@MSi, 30CaP@MSi composites (200–500-μm particle size) on osteoblasts viability was evaluated by 2D collagen gel test (Collagen I, Rat tail, Sigma-Aldrich). The commercial hydroxyapatite (Hap) was tested for comparative purposes. Before the assays, all tested materials were sterilized by heating in air for a period of 3 h at 300 °C in a muffle furnace (M-525, II series). Each material was suspended in collagen solution at three tested concentrations: 10, 100 μg/mL, and 1 mg/mL, then vortexed, alkalized, and applied into each well of 48 well plate in the volume of 150 μL for gelation. After 30 min of incubation at 37 °C, osteoblasts were seeded at 2.5 × 10^4^ cells on collagen gels in each well and incubated for 72 h. Cells cultured onto collagen gel without suspended material was studied as a control. Fluorogenic esterase substrate, BCECF-AM, was used for visualization and quantitative evaluation of viable cells. This ester is passively loaded into viable cells, and converted by intracellular esterases into fluorescent products. BCECF-AM staining was performed according to the standard protocol. Images were obtained with a Axiovert 200 microscope equipped with AxioCam MRm digital camera (Zeiss, Thornwood, NY). For quantitative evaluation of cells viability, fluorescence was measured with excitation/emission at 439/535 nm using Synergy H1 microplate reader (BioTek, Winooski, VT). For fluorescence-based quantification, fluorescence at 535 nm for the background (collagen gel with suspended material and culture medium without cells) was subtracted from each cell sample.

Data was presented as the mean ± standard deviations for three independent experiments. Statistical analysis was performed by Student’s *t* test using STATISTICA 13.3 software (Statsoft, Poland). The results were considered to be statistically significant when *p* value was < 0.05 vs control.

### Formulation of drug delivery systems

The optimal CaP@MSi composite characterized by the highest mineralization potential and relatively low cytotoxicity against osteoblasts was used for drug adsorption and pelletization process.

For the drug-loading experiment, the doxycycline hydrochloride (DOX, Sigma-Aldrich) was used as the model, water soluble antibiotic. For the drug-loading experiment, the immersion procedure was applied as described in our previous reports [19, 20]. Briefly, DOX was first dissolved in purified water (10 mg/mL). The optimal composition of CaP@MSi (200–500 μm particle size) was then added to the solution and the suspension was stirred for at least 2 h at 25 ± 0.5 °C (protected from light) to ensure the equilibrium adsorption state. Each 500 mg of CaP@MSi powders was suspended in 10 mL of DOX solution. The suspension was next filtrated under vacuum and the concentration of the DOX remaining in the solution was examined spectrophotometrically by monitoring the changes in absorbance at 347 nm—DOX analytical wavelength in aqueous medium (UV-Vis spectrophotometer Shimadzu, model UV-1800). The DOX-loaded CaP@MSi composites (DOX-CaP@MSi) were dried at room temperature for 24 h. The amount of DOX adsorbed onto the CaP@MSi composite and adsorption efficiency were calculated using Eqs. 2 and 3, respectively:

2 $$ {Q}_e=\frac{\left({C}_0-{C}_e\right)\bullet V}{m} $$

3 $$ {\%}_{Ads}=\left(\frac{C_0-{C}_e}{C_0}\right)\bullet 100\% $$

where Q_e_ (mg/g) is an amount of DOX adsorbed at the equilibrium state, %Ads (%) is an adsorption efficiency, C_0_ (mg/mL) is an initial DOX concentration, C_e_ (mg/mL) is DOX concentration at equilibrium state, V (mL) is a volume of DOX solution, and m (g) is the mass of CaP@MSi composite. The mean adsorption efficiency with standard deviation were also calculated.

The pellets based on the DOX-CaP@MSi composite were prepared by the wet granulation, extrusion, and spheronization technique using Caleva Multi Lab apparatus. Based on the preliminary studies, the pellets with both the satisfactory mechanical properties and 50 wt% of calcium phosphate-mesoporous silica composite were obtained using the composition given below. The CaP@MSi and DOX-CaP@MSi were used in ratio of 38 and 12 wt%, respectively. The 12 wt% of DOX-CaP@MSi in the formulation corresponded to the 2 wt% content of drug similar to drug content in commercially available spherical granules (0.5–5 wt%) [21, 22]. The microcrystalline cellulose (MCC; Avicel PH 101, Sigma-Aldrich) and ethyl cellulose (EC; Ethocel 20 cP, Dow Chemical) were used as the excipients (45 and 5 wt%, respectively). The batch size of each batch was 5 g. Briefly 1.90, 0.60, 2.25, and 0.25 g of CaP@MSi, DOX-CaP@MSi, MCC, and EC were used, respectively. The 0.60 g of DOX-CaP@MSi corresponded to 100 mg of DOX providing 2 wt% drug content in the formulation. Powders were accurately weighed, premixed in a mortar, and homogeneously mixed in a granulator attachment (100 rpm, 5 min) and then mass was wet-agglomerated using EC ethanolic binder solution (5 wt%) in the same attachment (100 rpm, 5 min). The optimal volume of binder solution, necessary for granulation, was determined in preliminary studies using Caleva Torque Rheometer. The wet mass was then extruded in an extruder attachment running at 100 rpm with a circular 1-mm holes diameter and depth. The entire batch of extrudate was then spheronized in a spheronizer attachment of diameter 8.5 cm (2000 rpm, 5 min). The resultant pellets were left to dry overnight at ambient conditions. The mean yield of the pelletization process (calculated as the ratio of total weight of dried pellets to initial weight of all used materials) was 80 ± 2%. For further studies (drug release and mineralization potential studies), the main fraction of pellets (0.8–1.0 mm) was chosen as the fraction obtained from sieving with the highest weight (≥ 90%). The actual amount of DOX in the pellets was determined by crushing the main fraction of the pellets and immersion in purified water with vigorous shaking (500 rpm, 24 h, room temperature) providing sink conditions. The total amount of DOX released from main fraction of the pellets was examined spectrophotometrically (by monitoring the changes in absorbance at 347 nm). The procedure was repeated for 3 batches.

### Release studies of DOX-CaP@MSi pellets

The drug release studies were performed using USP II Paddle Apparatus (Copley DIS-6000) at 37.0 °C, 50 rpm. Briefly, the 3.8 g of the pellets, 0.8–1.0 mm fraction (equivalent to 72 mg of adsorbed DOX) was used in each test. A constant fraction of the pellets was used for each batch in order to minimize the effect of the change in total surface area of the pellets on the drug dissolution rate. Phosphate buffer (pH = 7.4; 500 mL) was used as the dissolution media providing sink conditions. At suitable time intervals, 2.0 mL of solutions was filtered using membrane filters (0.22 μm) and analyzed spectrophotometrically at 352 nm—DOX analytical wavelength in phosphate buffer medium. Analytical studies were conducted in accordance with the requirements for quantitative analyses, calibrating the detector with a standard solution of the tested DOX in buffer. The drug stability was provided during the whole release studies. Drug release data were plotted as the cumulative percent of DOX released (Q) as a function of time (t). The release studies were repeated 6 times. The same release studies were carried out for pristine DOX-CaP@MSi powders (with 72 mg of adsorbed DOX) for comparative purposes.

### In vitro mineralization assay of DOX-CaP@MSi pellets

The mineralization potential studies of DOX-CaP@MSi pellets were carried out following similar procedure as described for CaP@MSi composites, except that 2 mg of pellets was used for each 1 mL of SBF. The SEM-EDX analysis was carried out for pellets before and after every 7 days of incubation in SBF. The SBF was exchanged every 24 h in accordance with reference [18]. Due to the sedimentation of the pellets, the SBF was exchanged by simple decantation method instead of centrifugation which was performed for CaP@MSi powders.

## Results and Discussion

This study is part of our ongoing effort to evaluate the ability of hexagonally ordered mesoporous silica, MSi, for two biomedical applications: local antibacterial drug delivery and bone tissue regeneration. We synthesized the biphasic composites of CaP with MSi to improve mineralization properties of parent 100MSi. *Rim et al*. [13] have already combined the CaP and the MSi developing absorbable, pH-tunable calcium phosphate covered MSi nanocontainers for intracellular controlled release of doxorubicin. They have emphasized that CaP is widely used as a bioactive osteoconductive coating for bone-regenerative materials which is formed after immersion of the material in SBF. They have used the CaP coating as pore blocker of drug-loaded MSi nanoparticles. Such coating may be dissolved in acidic cellular environments to provide the release of entrapped doxorubicin directly in the target tumor cells. In our case, we would like to obtain the CaP@MSi composites in the form of 1 mm spherical granules—pellets which after implantation during the surgery may act as bifunctional, local bone drug delivery system: (i) releasing the drug directly in the infected area and (ii) regenerating the bone defects.

In the unpublished results, we performed pre-optimized selection studies with different amounts of CaP in MSi (up to 50%) to find optimal CaP@MSi composition. We were taking into account 3 parameters of obtained composites: porosity, adsorption process of DOX (both the adsorption efficiency and repeatability), and mineralization potential. We found that the addition of CaP higher than 30% resulted in disordering of the MSi@CaP structure. The disordering of MSi@CaP structure caused the unrepeatable and also relatively low adsorption of DOX. The reason of observed disordering is the consequence of the precipitation of calcium phosphates particles and their aggregation in the mesoporous silica network. These all heterogeneous CaP-rich regions may induce local network deformations responsible for a decrease in bulk porosity of the MSi@CaP. Moreover, in the mineralization studies, we also found that the rate of mineralization for composites with CaP above 30% was not significantly higher compared to 30CaP@MSi. Moreover, those composites containing higher than 40% of CaP showed tendency to phase separation. These formulations had been easily disintegrated in the distal portion of small particles in simulated body fluid.

In the next step of studies, we selected the formulation of CaP@MSi characterized by highest mineralization potential to load the model antibiotic—doxycycline (DOX) by adsorption process and prepare drug delivery system in the form of spherical granules (pellets) considering the cytotoxicity values of CaP@MSi composites. During the pelletization process, we used only these pharmacopeial excipients which are known to be non-toxic and biocompatible [23, 24].

### Characterization of synthesized CaP@MSi

**Fig. 1:**
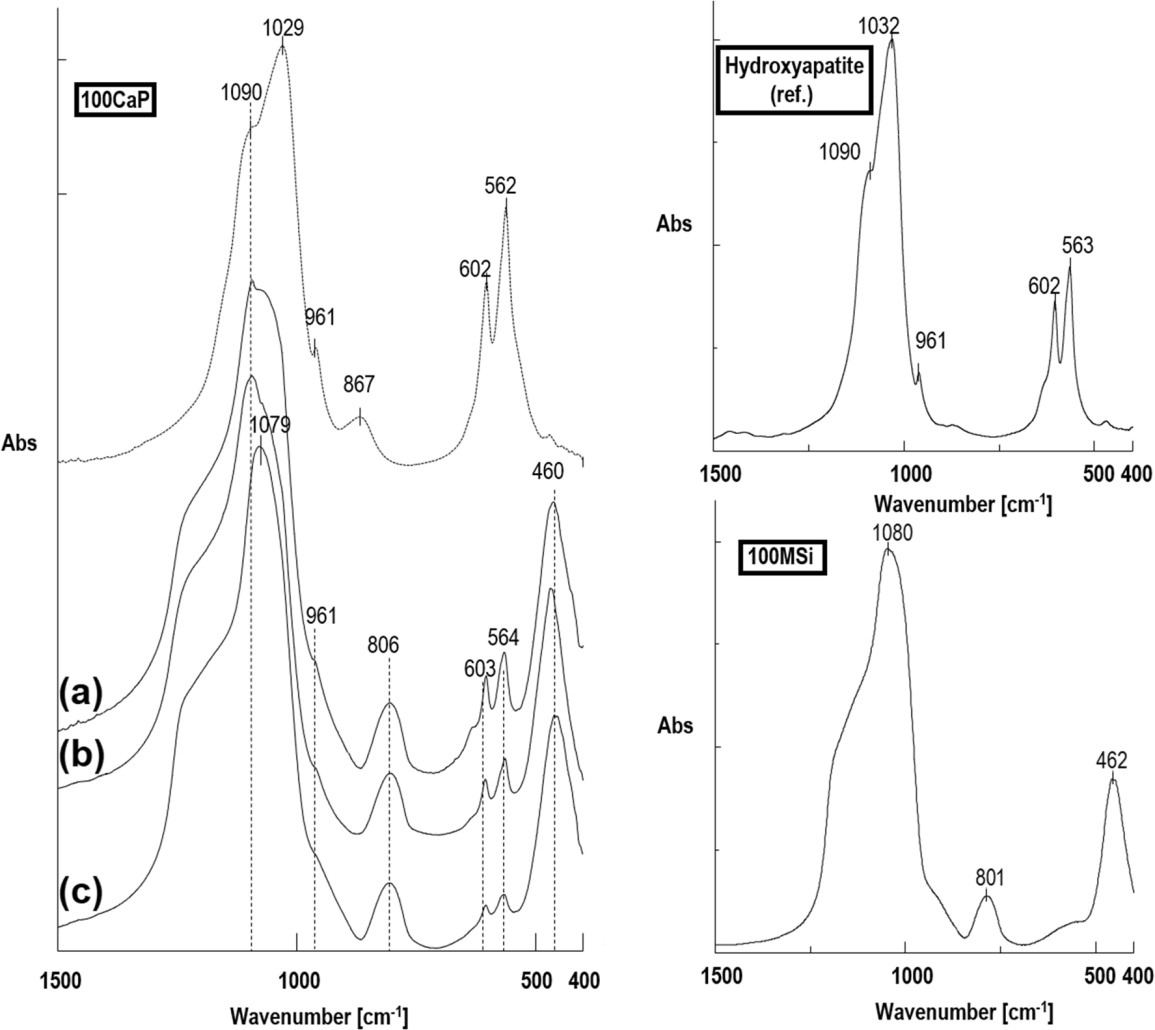
FTIR spectra of synthesised materials: 100CaP, 30CaP@MSi (a), 20CaP@MSi (b), 10CaP@MSi (c), 100MSi and hydroxyapatite reference sample

**Fig. 2:**
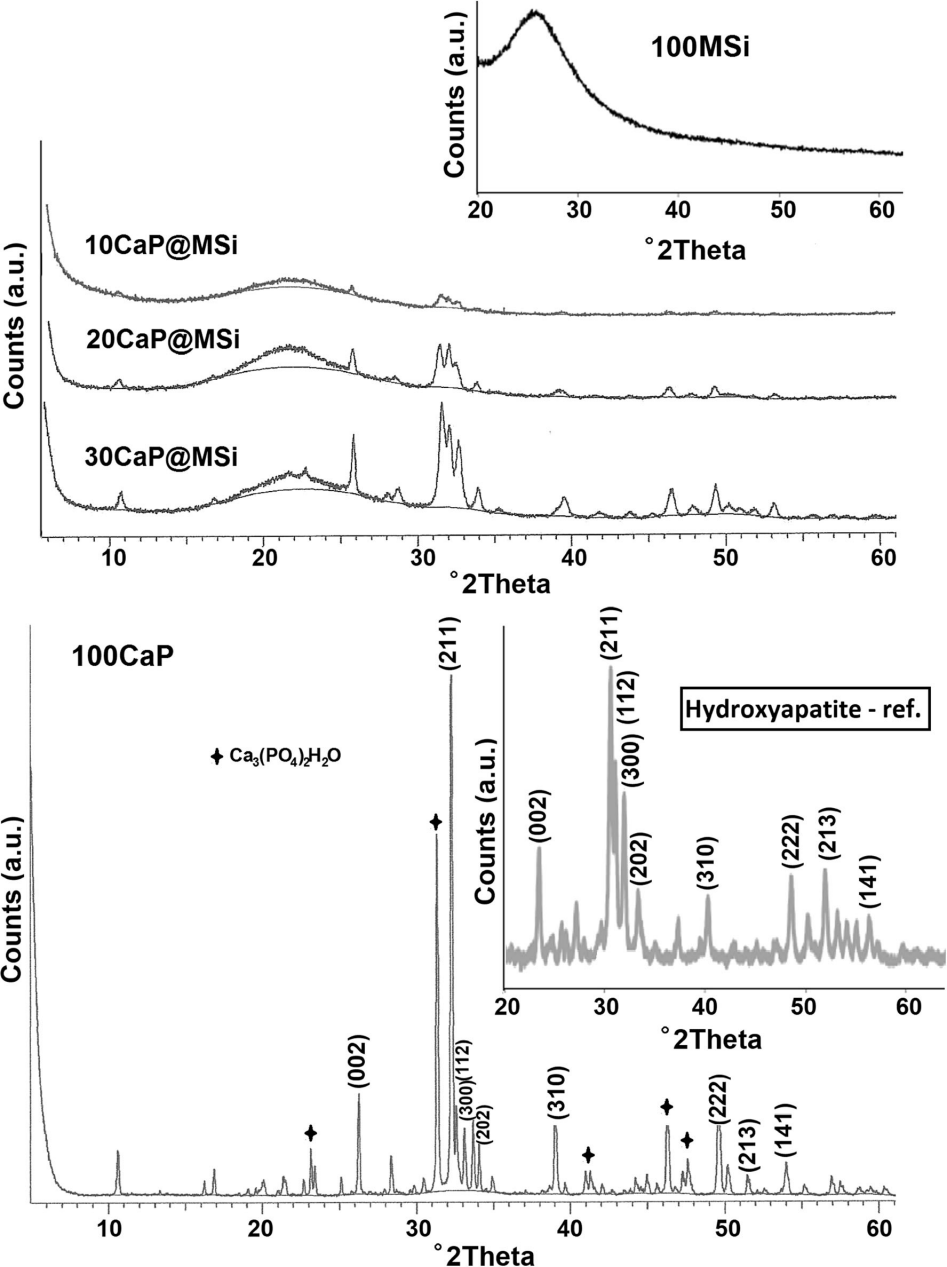
XRD patterns of synthesised materials: 100MSi, 10CaP@MSi, 20CaP@MSi, 30CaP@MSi, 100CaP and hydroxyapatite reference sample

**Fig. 3:**
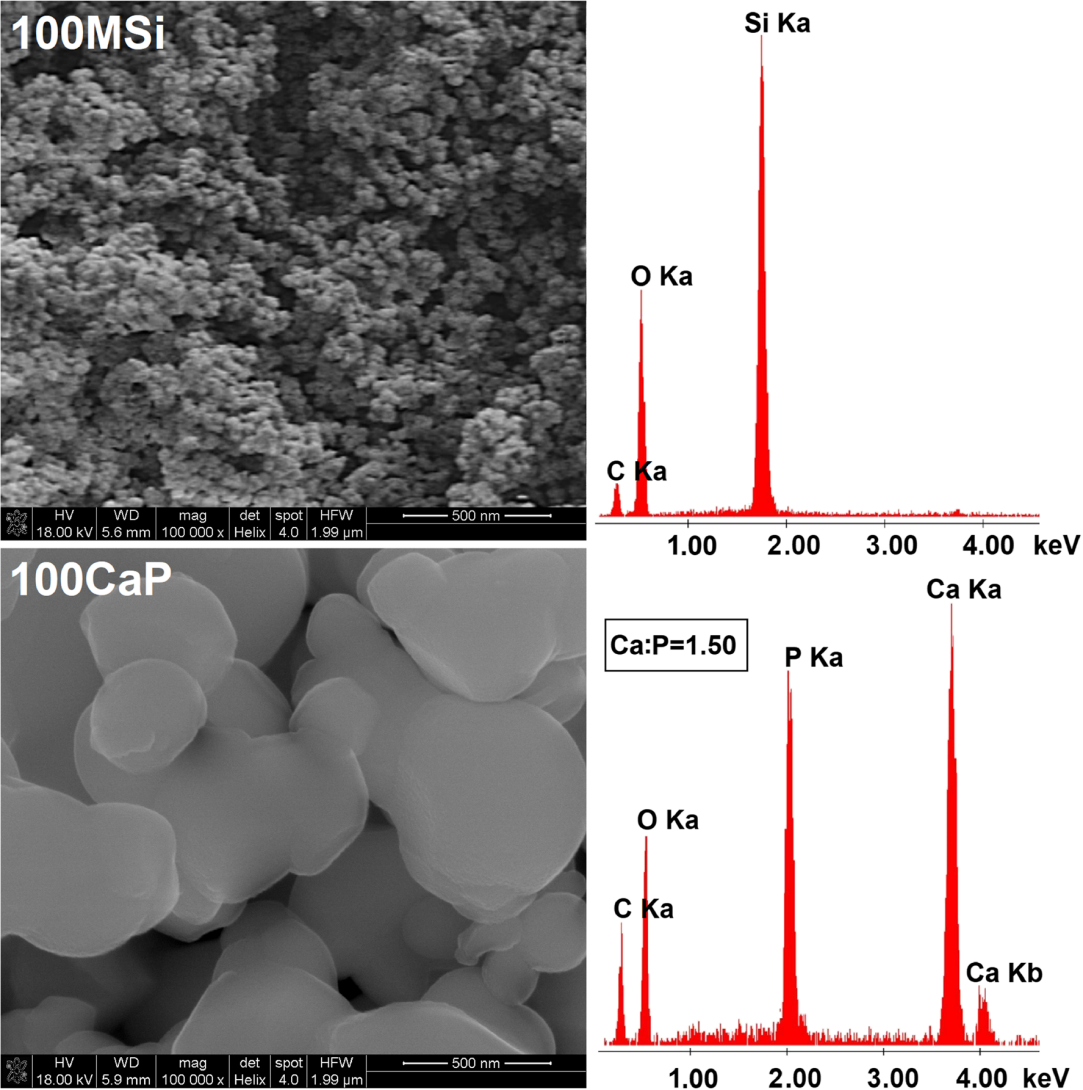
SEM-EDX micrographs of synthesised materials: 100MSi and 100CaP

**Fig. 4:**
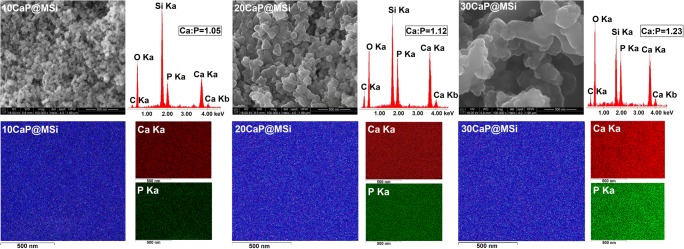
SEM-EDX micrographs of synthesised materials: 10CaP@MSi, 20CaP@MSi, 30CaP@MSi with corresponding EDX-mapping: Si-blue, Ca-red, P-green

The detailed FTIR spectra of the as-synthesized CaP@MSi composites are available in Supplementary material 1. The FTIR results confirmed both the successful removal of CTAB during the calcination process and the presence of phosphate groups in the composites after synthesis. Figure 1 shows the comparison of FTIR spectra between a commercial hydroxyapatite (Hap) and as-synthesized 100CaP, parent 100MSi, and CaP@MSi samples. The FTIR spectra for Hap and 100CaP were almost the same, only the new peak at 867 cm^−1^ (ν2) of carbonate group was observed in the 100CaP sample. This band was not clearly distinguished for the CaP@MSi composites and it could also overlap together with a Si-O-Si stretching mode at 800 cm^−1^ from the MSi. In the Hap and 100CaP spectra, the peaks at 603 cm^−1^ and 564 cm^−1^ were assigned to (PO_4_)^3-^ groups with vibrational mode of ν4 whereas the peaks at 1090, 1032, and 961 cm^−1^ were characteristic for the ν3, ν3, and ν1 vibrational modes of (PO_4_)^3-^, respectively [25]. The peaks at 603 cm^−1^ and 564 cm^−1^ proved that the CaP phase in CaP@MSi composites was successfully formed during the templating method: with the increase of CaP in MSi the intensities of 603 cm^−1^ and 564 cm^−1^ peaks increased. For the FTIR spectrum of composite with the largest amount of CaP (30CaP@MSi), the 1090 cm^−1^ was also observed. Figure 2 shows the PXRD patterns of the pure 100CaP, parent 100MSi, and CaP@MSi composites (10CaP@MSi, 20CaP@MSi, 30CaP@MSi). The typical broad diffraction halo was observed for the parent 100MSi. In contrast, phase analysis showed that all major peaks of hydroxyapatite were present in the as-synthesized pure 100CaP. The presence of low crystalline apatite (Ca_3_(PO_4_)_2_·H_2_O), typical hydrated calcium phosphate phase, was also observed in the XRD patterns of the 100CaP. This result has been also identified by other authors that used the wet precipitation method for the CaP synthesis [26]. In the all CaP@MSi composites, the amorphous halo characteristic for the parent 100MSi phase was observed. The CaP identification in the CaP@MSi composites was based on the presence of a diffraction peak at 2θ = 26° and a broad diffraction feature in the region of 2θ = 31–34°. The lowest intensity of these peaks was observed for 10CaP@MSi and with the increase of CaP in MSi the doublet of diffraction peaks at 2θ = 31° became partially resolved. These diffraction peaks could also indicate the presence of low crystalline calcium phosphate mineral in the CaP@MSi composites compared to the as-synthesized 100CaP. The results indicated that the crystallinity of CaP was reduced with increasing content of MSi in composite, indicating that MSi could cause alteration of the CaP structure. This suggests that MSi could control the particle dimension of CaP particles and could prevent the aggregation as well as the crystallization of CaP. This might be attributed to the molecular motion and interfacial kinetics which limit the growth of crystal and therefore the crystallite size of CaP at the presence of MSi. It was also confirmed by SEM analysis. Figure 3 shows the SEM images with the representative EDX profile of the morphology of pure 100CaP and parent 100MSi. Supplementary material 2 shows the TEM image of representative particle of 100CaP and 100MSi. For the 100CaP, the homogenous rode-like structures were obtained. For the parent 100MSi, the homogenous surface of spherical particles was observed. The measured average particle size of 100CaP and 100MSi was 370 ± 50 nm and 100 ± 12 nm, respectively (Supplementary material 2). Figure 4 shows the SEM images with the representative EDX profile and mapping of CaP@MSi composites (10CaP@MSi, 20CaP@MSi, 30CaP@MSi). For the 10CaP@MSi, and 20CaP@MSi, the spherical-shaped phase was still observed with tendency to agglomeration that led to the larger particle size for 20CaP@MSi. Whereas for 30CaP@MSi, the elongated, rod-like-shaped structure was observed. The smallest spherical particles of CaP precipitated in MSi (average particle size was 20 ± 8 nm) were obtained for the lowest (10%) content of CaP in MSi, whereas the largest rod-like-shaped particles (average particle size was 47 ± 14 nm) were obtained for the largest (30%) content of CaP in MSi. This was also confirmed by TEM results (Supplementary material 2). The representative TEM images of 30CaP@MSi and parent 100MSi evidently indicated the biphasic structure of some rod-like elongated particles embedded in the ordered hexagonal structure of MSi. A study done by *Santos et al.* [27] has shown that precipitated Hap particles can be characterized by different shapes such as long columns, thick-like plates, and needle-like rods if the precipitation reaction is carried out at alkaline reactions by using Ca(OH)_2_ and H_3_PO_4_ as the starting materials.

In summary, the microscopic and PXRD results have indicated that MSi can control the particle size of CaP phase and partially prevent the aggregation as well as the crystallization of CaP. The N_2_ adsorption-desorption isotherms data confirmed these observations as well. Supplementary material 3 shows the N_2_ adsorption-desorption isotherms (a) and the pore-size distribution (b) with the summarized data of BET specific surface area, the total pore volume and average pore diameter corresponding to the pure 100CaP, parent 100MSi, and CaP@MSi composites (10CaP@MSi, 20CaP@MSi, 30CaP@MSi). The N_2_ isotherms of pure 100CaP showed a type IV with the H3-type hysteresis characteristic for slits-shaped pores. This kind of pores has been often observed for plate-like structure [28]. Both the BET surface area closed to 128 m^2^/g and the broad distribution of pores with no clear peak in pore size distribution (Supplementary material 3) suggest almost the non-porous state of pure 100CaP. The N_2_ isotherms of parent 100MSi and 10CaP@MSi showed a type IV with a reversible isotherm for MSi and H1-type hysteresis for 10CaP@MSi, corresponded to cylindrical mesopores [29]. For the 20CaP@MSi and 30CaP@MSi, the adsorption isotherms were characterized by two separated steps, corresponding to N_2_ condensation in the MSi (at low pressure) and in the CaP domains (at high pressure). The broader distribution of pores with smaller pore diameter for CaP@MSi composites compared to the parent 100MSi indicated the heterogeneous nature of CaP@MSi composites due to the existing of two phases—MSi and CaP confirmed by SEM and TEM images (Fig. 4 and Supplementary material 2). From the analysis of the porosity, it could be claimed that pore diameter, pore volume, and specific surface area were representative of the individual porosities of both the 100CaP and parent 100MSi components. This means that with an increase in the amount of CaP in MSi, the specific surface area decreased by a factor of 3.0 (from 765 m^2^/g for the parent 100MSi to 250 m^2^/g for 30CaP@MSi), the total pore volume decreased by a factor of 2.3 (from 0.54 to 0.23 cm^3^/g, respectively) whereas the average pore diameter slightly increased (from 28 Å to 37 Å, respectively).

According to the complementary results from FTIR, PXRD, SEM, and nitrogen sorption analyses, it might be summarized that spontaneous formation of biphasic CaP@MSi composite is followed by two steps: (1) the dissolution of the K_2_HPO_4_, CaCl_2_, and TEOS at CTAB solution in ammonia medium (pH = 10) and (2) the composite self-assembly on the rod-like CTAB micelles. In details, the silanol anions of hydrolysed TEOS (SiO^-^) together with phosphate ions may neutralize the positively charged ammonium groups of CTAB and form CTAB^+^SiO^-^ and CTAB^+^PO_4_^3-^. These species can further interact with the Ca^2+^ from CaCl_2_. After removing of CTAB, the spherical-shaped SiO_2_ and plate-like-shaped CaP structures are produced and hence the biphasic composite structure is formed. The different Ca:P molar ratios of the precipitated CaP phases in MSi suggest that the MSi can effectively control both the particle size and the subsequent arrangement of CaP particles. In case of 100CaP, the steric hindrance is low; thus, the relatively large plate-like-shaped Hap was synthesized followed by the spontaneous growing of the CaP nuclei. With an increase in the MSi:CaP mass ratio, the number of CaP which may interact with SiO_2_ around the CTAB micelles decreases; hence, the additional steric hindrance is reinforced and the smaller CaP nuclei will grow. Thus, smaller rod-like-shaped CaP structures are observed in the presence of MSi.

### In vitro mineralization assay of CaP@MSi

**Fig. 5:**
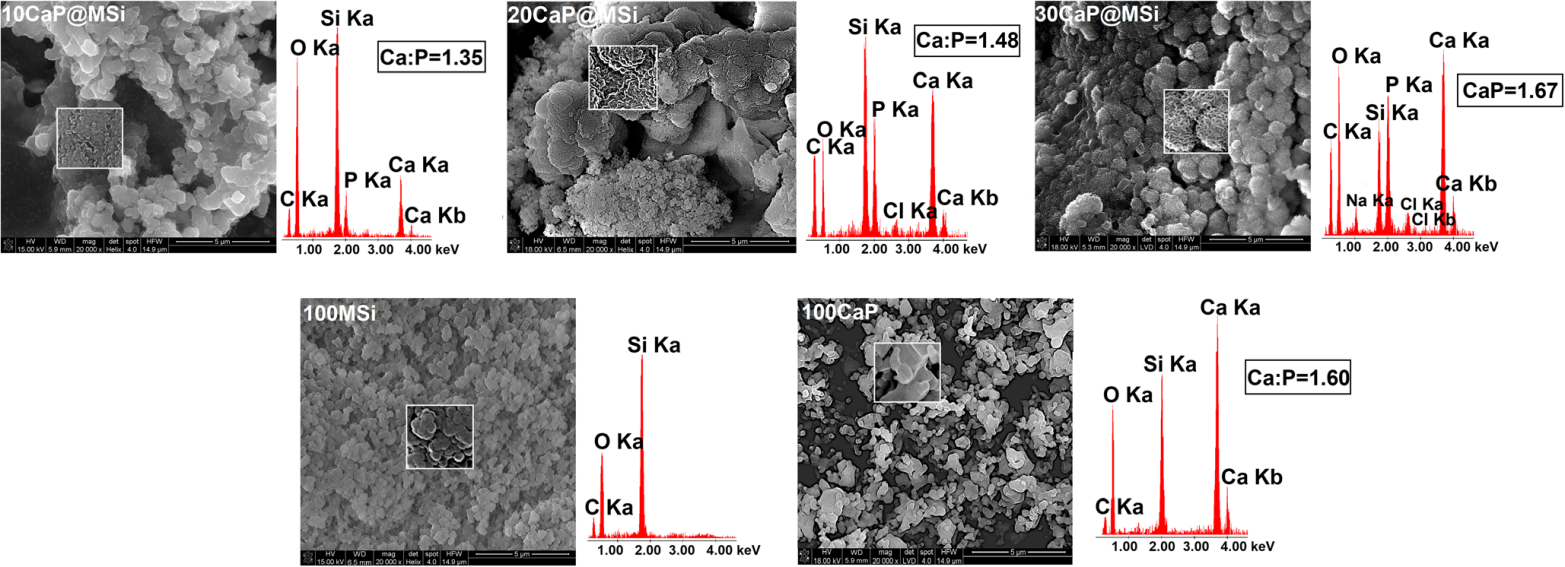
SEM-EDX micrographs of 10CaP@MSi, 20CaP@MSi, 30CaP@MSi, 100MSi and 100CaP materials after 7 days of incubation in SBF

To investigate the mineralization potential of the biphasic CaP@MSi composites, the representative samples of each composites were immersed in simulated body fluid for 28 days with daily exchange of SBF. The changes in the Ca:P ratios of CaP@MSi composites are presented in Supplementary material 4. After 7 days of incubation in SBF, the Ca:P ratio of 1.67, close to natural bone apatite [30], was only observed for the 30CaP@MSi composite, whereas the 20CaP@MSi and 10CaP@MSi composites required approx. 14 and 28 days, respectively. Figure 5 shows the representative SEM-EDX results for 10CaP@MSi, 20CaP@MSi, 30CaP@MSi composites and 100MSi, 100CaP samples after 7 days of incubation in SBF. For 10CaP@MSi, the random clusters of calcium phosphates appeared with Ca:P molar ratio of 1.35. For 20CaP@MSi, clusters transformed into continuous agglomerates (Ca:P = 1.48). SEM image showed that the surface of the 30CaP@MSi was completely covered by a calcium phosphate species with morphology characteristic for hydroxyapatite. The relatively higher content of carbon in EDX of 30CaP@MSi may suggest the incorporation of carbonates into hydroxyapatite structure. The mechanism of formation of Hap on the CaP@MSi may be related to the biomimetic behavior of CaP@MSi in simulated body fluid comparable with bioactive properties of bioglass [31]. A biomimetic apatite coating can grow on the CaP@MSi surface by attachment of Ca^2+^ and PO_4_^3-^ ions from SBF. However, it was found from SEM results that the surface morphology of 100MSi and 100CaP did not change after 7 days of incubation in SBF. Moreover, 100MSi sample exhibited the same EDX profiles before and after 7 days of mineralization assay, whereas for the 100CaP, the EDX results shown that Ca/P ratio slightly changed from 1.5 to 1.6. The lack of characteristic carbonated Hap semi-crystallites with Ca/P ratio around 1.67 [32] on the surface of 100CaP suggested that only some reorganization of the 100CaP structure in simulated body fluid took place. This could suggest that the 100CaP obtained by the wet precipitation method did not react with ions from the SBF, what was necessary to create a biomimetic carbonated apatite [33] or the reaction would take place in much slower manner compared to 30CaP@MSi. In summary, from these results, the highest mineralization potential was observed for the 30CaP@MSi composite.

### *In vitro* cytotoxicity of CaP@MSi

**Fig. 6:**
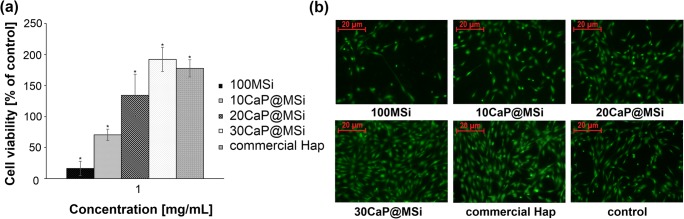
Evaluation of viability of osteoblasts grown onto collagen gels for 72 h with parent 100MSi, CaP@MSi, and commercial Hap (a); fluorescent images of live osteoblasts (BCECF-AM) (b). The values presented are means ± SD (n = 3). *Statistically significant, p < 0.05 vs control

To establish an impact of tested materials on osteoblasts viability, cells were exposed to collagen gel with suspended: parent 100MSi, 10CaP@MSi, 20CaP@MSi, 30CaP@MSi composites, and the commercial hydroxyapatite (Hap) (200–500-μm particle size) at concentrations of 10, 100 μg/mL, and 1 mg/mL, respectively. The cells cultured on bare collagen gel was used as control. There were no statistically significant differences in the cells viability from particular materials at lower concentrations (10 and 100 μg/mL) (results not shown). The results obtained from 2D collagen gel test for the highest concentration (1 mg/mL) were presented in Fig. 6(a). The cells incubated with parent 100MSi spread less and took on the spherical morphology indicating its low biocompatibility (Fig. 6(b)). It probably results from the well-known high adsorptive properties of mesoporous silica materials that can contribute to adsorption of essential components of cell culture medium (e.g., amino acids, growth factors) [34, 35], thereby weakening cell viability. On the other hand, 30CaP@MSi presented influence on the cell viability similar to the commercial Hap with proven biocompatibility towards osteoblasts [36]. Moreover, the viability of cells cultured on collagen gel with suspended 30CaP@MSi increased twofold in relation to control. The higher content of CaP in composite, the better spreading, adhesion and more elongated, flattened morphology of cells was presented (Fig. 6(b)). To conclude, the results obtained from the 2D collagen gel test suggest that the biocompatibility of CaP@MSi composites at higher concentration (1 mg/mL) seems to be positively correlated with the tested CaP content in MSi composites. According to the literature, calcium is a critical factor in osteoblast proliferation, survival, differentiation, and mineralization [37, 38]; thus, the observed correlation and high value of cell viability for 30CaP@MSi may be related to calcium release from composites into the cell culture medium.

### Pellets based on the DOX-30CaP@MSi

Based on the presented mineralization potential studies and the *in vitro* cytotoxicity tests, the 30CaP@MSi composite was chosen for DOX adsorption and pelletization process because it was characterized by the highest mineralization potential and low cytotoxic effect on the osteoblasts cells.

### DOX adsorption onto the 30CaP@MSi

The adsorption efficiency was 84 ± 3% what corresponds to 165 ± 9 mg of DOX adsorbed per 1 g of 30CaP@MSi. The high adsorption efficiency in the performed adsorption conditions (pH = 3.5) might be explained by the favorable interactions between amine group present in DOX molecules and silanol groups present on the 30CaP@MSi composite surface. At pH = 3.5, the amine group of DOX molecule exists in the protonated form (R_3_NH^+^; pKa = 9.7) [39] whereas the free silanols (≡SiOH) of 30CaP@MSi are dissociated (≡SiO^-^; pKa = 2.0) [40]. Thus, the ionic attraction between DOX molecules and 30CaP@MSi surface may occur. Additionally, the other polar groups of the DOX molecule (phenolic, amide, carbonylic) could interact with both the dissociated free silanols and undissociated geminal silanols (=Si(OH)_2_, pKa = 8.0) [40] of 30CaP@MSi surface *via* hydrogen bonds, thus increasing the adsorption efficiency. The drug adsorption onto the 30CaP@MSi was confirmed by FTIR (Supplementary material 5) where the vibrations specific for DOX were being observed in the ranges of 3000–2800, 1600–1100, and 950–600 cm^−1^ for DOX-30CaP@MSi sample. The peaks characteristic for the polar groups of the antibiotic were shifted to lower frequencies respect to DOX reference spectrum what confirmed the interactions between drug molecules and 30CaP@MSi surface.

### Morphology of the DOX-30CaP@MSi pellets

**Fig. 7:**
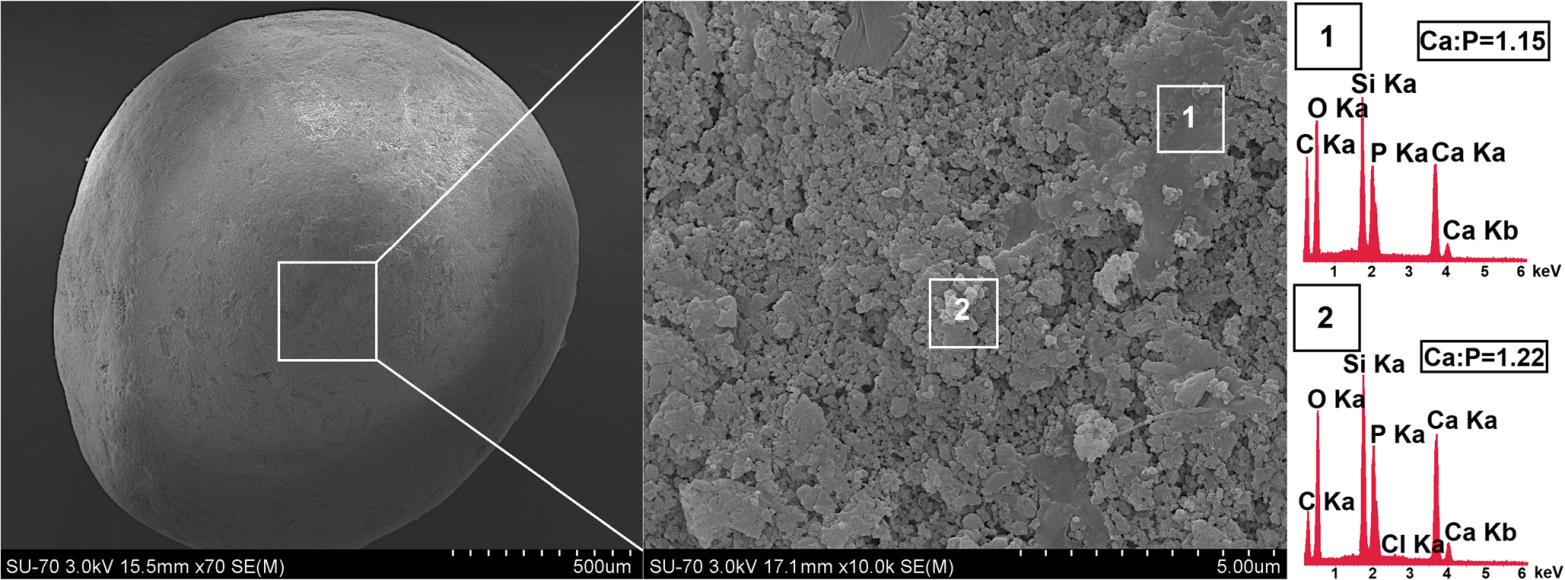
SEM-EDX micrograph of DOX-30CaP@MSi pellet

DOX-30CaP@MSi pellets were successfully prepared by wet granulation, extrusion, and spheronization method. The drug-loaded pellets (Fig. 7) were found to be spherical with satisfactory physical properties which allowed for further studies (drug release and mineralization potential studies) without risk of pellets cracking. As shown in Fig. 7, the pellets surface was composed of continuous, organic regions made of microcrystalline and ethyl cellulose (relatively high carbon content observed in EDX) and heterogeneous, inorganic regions made of biphasic 30CaP@MSi composite. The presence of chloride in EDX profile no. 2 (Fig. 7) derives from DOX molecules adsorbed onto the 30CaP@MSi composite accordingly to FTIR results (Supplementary material 5).

### DOX release studies

**Fig. 8:**
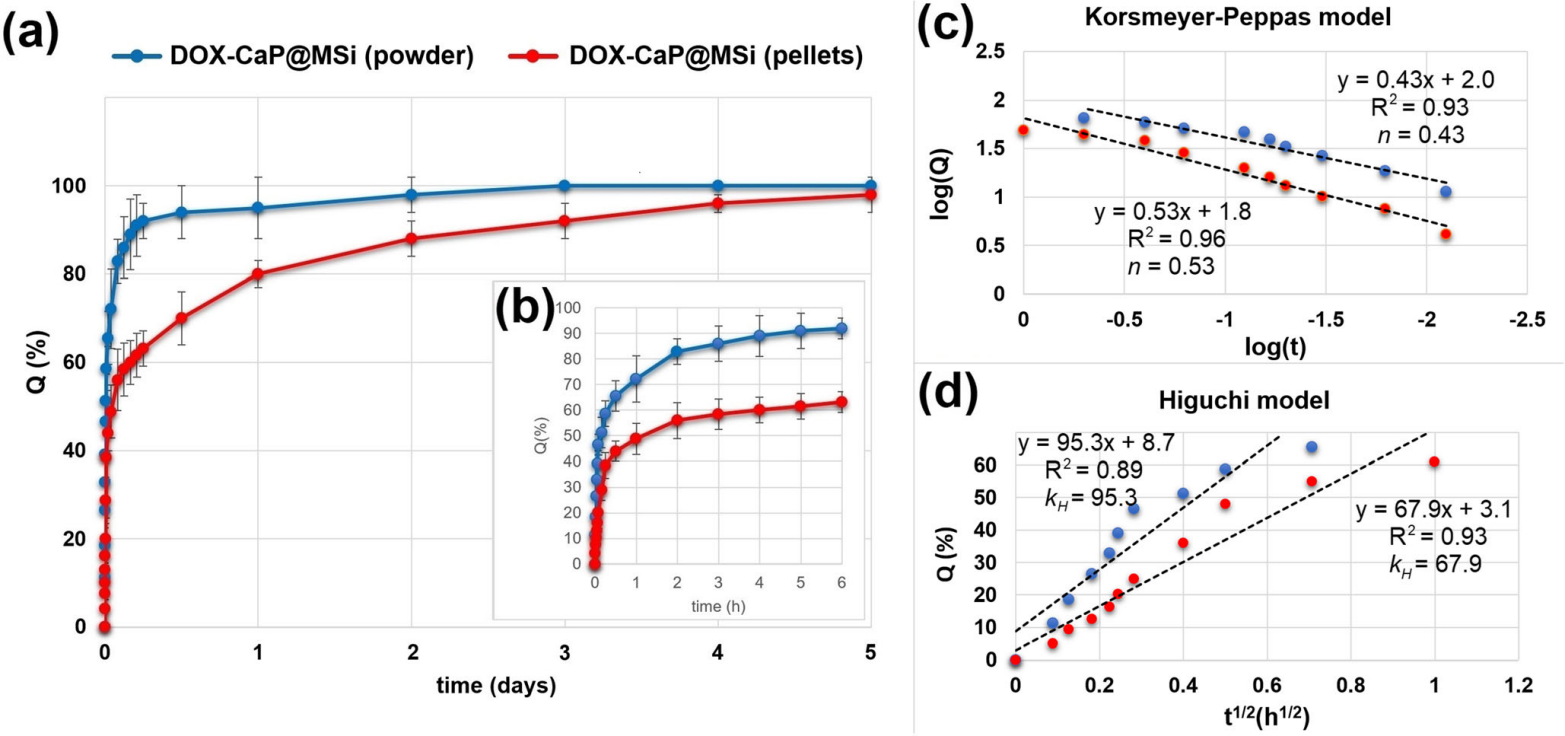
Release profiles of DOX-30@CaP@MSi powders (blue) and pellets (red): total drug release profile (a); first 6 h of drug release (b); Korsmeyer-Peppas model (c); Higuchi model (d). The values presented are means ± SD (n=3); n–release exponent in Korsmeyer-Peppas model, k_H_–Higuchi dissolution constant (h^-1/2^), R^2^–coefficient of determination

Figure 8 shows the release profiles of DOX for both the DOX-30CaP@MSi powders and pellets. The cumulative percent of DOX released (Q) as a function of prolonged time (Fig. 8(a)) and initial 6-h burst release (Fig. 8(b)) are presented. A significant burst effect of the DOX release from the DOX-30CaP@MSi powders was observed, about 90 ± 7% of adsorbed drug was being released in the first 4 h of studies (Fig. 8(b)) with complete drug release after 2 days (Fig. 8(a)). In the case of DOX-30CaP@MSi pellets, the burst release was reduced by a factor of 1.5, only 60 ± 4% of drug was released after 4 h (Fig. 8(b)). For DOX-30CaP@MSi pellets, the drug release was prolonged to 5 days.

For the DOX-30CaP@MSi powders, the high burst release of DOX is observed most probably due to weak interaction between DOX and silica surface. Similar observations for DOX-loaded MSi were noticed by *Deacuno et al.* [41] who declared, that initial burst release of DOX has been strongly associated with the type of functional groups presented on MSi surface. *Ide et al.* [42] claimed that the calcination process of MSi results with the low concentration of surface silanol groups. Therefore, low concentration of silanols impedes the composite-DOX interactions (ionic, hydrogen bonding) which are crucial for prolonged drug delivery systems [19, 20]. In case of DOX-30CaP@MSi powders, the high burst release was also the result of DOX location (at least in part) out of the pores what is characteristic for the performed adsorption method (adsorption from concentrated solution). The DOX fraction presents loosely on the external silica surface dissolved immediately after immersion in release medium.

For the DOX-30CaP@MSi pellets, the reduction in DOX burst release with simultaneous prolongation of total drug release was achieved due to formation of spherical matrix (pellets) insoluble in release medium with increased hydrophobicity because of using of ethyl cellulose as a filler and binder. The release profile of DOX-30CaP@MSi pellets was characterized by two stages (Fig. 8(a) and (b)): first stage—an initial 50% release of loaded DOX in 1 h and second stage with prolonged drug release during 5 days. Such two-stage drug release profile of pellets seems to be a promising feature in current strategies of osteomyelitis treatment which focuses on both the reduction of systemic dosage and the increase of the antibiotic concentration directly in bone tissue [43]. On the one hand, the relatively high initial release of DOX during first 24 h might be considered as a loading dose at the beginning of the treatment. For example, *Trizio et al*. [44] obtained gentamycin-loaded eumelanin nanoparticles as potential bone drug delivery system in which the complete drug release was observed after 24 h with high burst release (approx. 72% in 1 h). Shorter, 4-h complete drug release was reported by *Dorati et al*. [45] who proposed gentamycin-loaded chitosan thermosetting hydrogels. In both publications, it has been claimed that obtained systems in vitro could exhibit potential antibacterial effect in the first stage of osteomyelitis treatment, due to the high local drug concentration provided in fast manner. On the other hand, the prolonged 5-day release of DOX from DOX-30CaP@MSi pellets might provide the maintaining dose of drug at infected bone sites. Approx. 5-day release of drugs was reported for both biodegradable and non-biodegradable bone delivery systems loaded with gentamycin, fosfomycin, vancomycin, ceftazidime, and tobramycin proving their potential ability to support the systematic treatment of osteomyelitis [46, 47]. Additionally, the complete drug release from proposed DOX-30CaP@MSi pellets is also an important feature of candidate for bone drug delivery system. Inert carriers such as bone cements may result in long (> 30 days) but incomplete drug release, causing a persistent release of sub-inhibitory concentrations of loaded antibiotics and increases the risk of antibiotic resistance [48].

Drug release from a porous matrix involves the penetration of release medium, drug dissolution, and leaching out of the drug through system of channels and pores. Based on the pharmaceutical technology of solid dosage forms [49, 50], the polymer content (microcrystalline cellulose, ethyl cellulose) increases both the matrix tortuosity and drug diffusion path-length and thus slows down the drug diffusion rate and release from the matrix. To prove these claims, the release kinetics studies have been performed. For spherical particles with a granular matrix containing a water soluble drug, the release kinetics may be described using Kosmeyer-Peppas or Higuchi models [20, 51]. Therefore, the release kinetic parameters for both the DOX-30CaP@MSi powders and pellets were calculated using linearized forms of Korsmeyer–Peppas and Higuchi models as presented in Eqs. 4 and 5, respectively.

4 $$ \mathit{\log}\ Q=n\ \mathit{\log}\ t+\mathit{\log}\ k $$

5 $$ Q={k}_H{t}^{\frac{1}{2}} $$

where Q denotes the fraction released by time t (h), *n* is an exponent related to the drug release mechanism, *k* (h^−n^) is a rate constant, and *k*_*H*_ is a Higuchi dissolution constant (h^-1/2^). In Eq. 4, *n* = 0.43 indicates a Fickian diffusion for spherical particles, and *n* = 1 corresponds to zero-order release. To find out the mechanism of drug release, the data for first 60% of drug release fraction (Q) were fitted with both models.

The kinetic parameters of fitted experimental data for DOX release with Korsmeyer-Peppas and Higuchi kinetic release models are presented in Fig. 8(c) and (d), respectively. For both the DOX-30CaP@MSi powders and pellets, the release kinetics were well characterized by Korsmeyer-Peppas model with *R*^2^ = 0.93 and 0.96, respectively (Fig. 8(c)). In case of DOX-30CaP@MSi powders, the release profiles were characterized by simple Fickian diffusion (*n* = 0.43), whereas for DOX-30CaP@MSi pellets the *n* value was found to be 0.53. The observed increase in *n* value (from 0.43 to 0.53) suggests the deviations in simple diffusion for DOX release from the pellets. If a dissolution medium penetrates into the pellets, then structural swelling, stress fields or matrix erosion may affect diffusion. The partial erosion/swelling of the pellets surface was also observed in SEM micrographs after drug release (Supplementary material 6). It is noteworthy, that the pellets matrix may also act as a hydrophobic boundary that impedes the access of solvent to drug molecules inside the matrix. Such boundary in case of DOX-30CaP@MSi powders is extremely lower and limited only to wall thickness of mesoporous silica material.

The drug release for both the DOX-30CaP@MSi powders and pellets was characterized by the profile less consistent with the Higuchi (*R*^2^ = 0.89 and 0.93, respectively) (Fig. 8(d)). For the DOX-30CaP@MSi powders, it is caused most probably by the DOX fraction present on the externally silica surface. This fraction dissolved rapidly after exposure to solvent. In case of DOX-30CaP@MSi pellets, the deviations from Higuchi model may be a consequence of the importance of excipient properties. While a pellets matrix is partially eroding or swelling, Eq. 5 should not be developed with stationary boundary conditions. However, the release kinetics for the DOX-30CaP@MSi pellets was characterized by relatively lower values of Higuchi dissolution constant (*k*_*H*_ = 67.9) (Fig. 8(d)) in comparison with DOX-30CaP@MSi powders (*k*_*H*_ = 95.3), indicating reduced burst release of DOX, what is in good correlation with experimental release profiles (Fig. 8(b)).

### *In vitro* mineralization of DOX-30CaP@MSi pellets

**Fig. 9:**
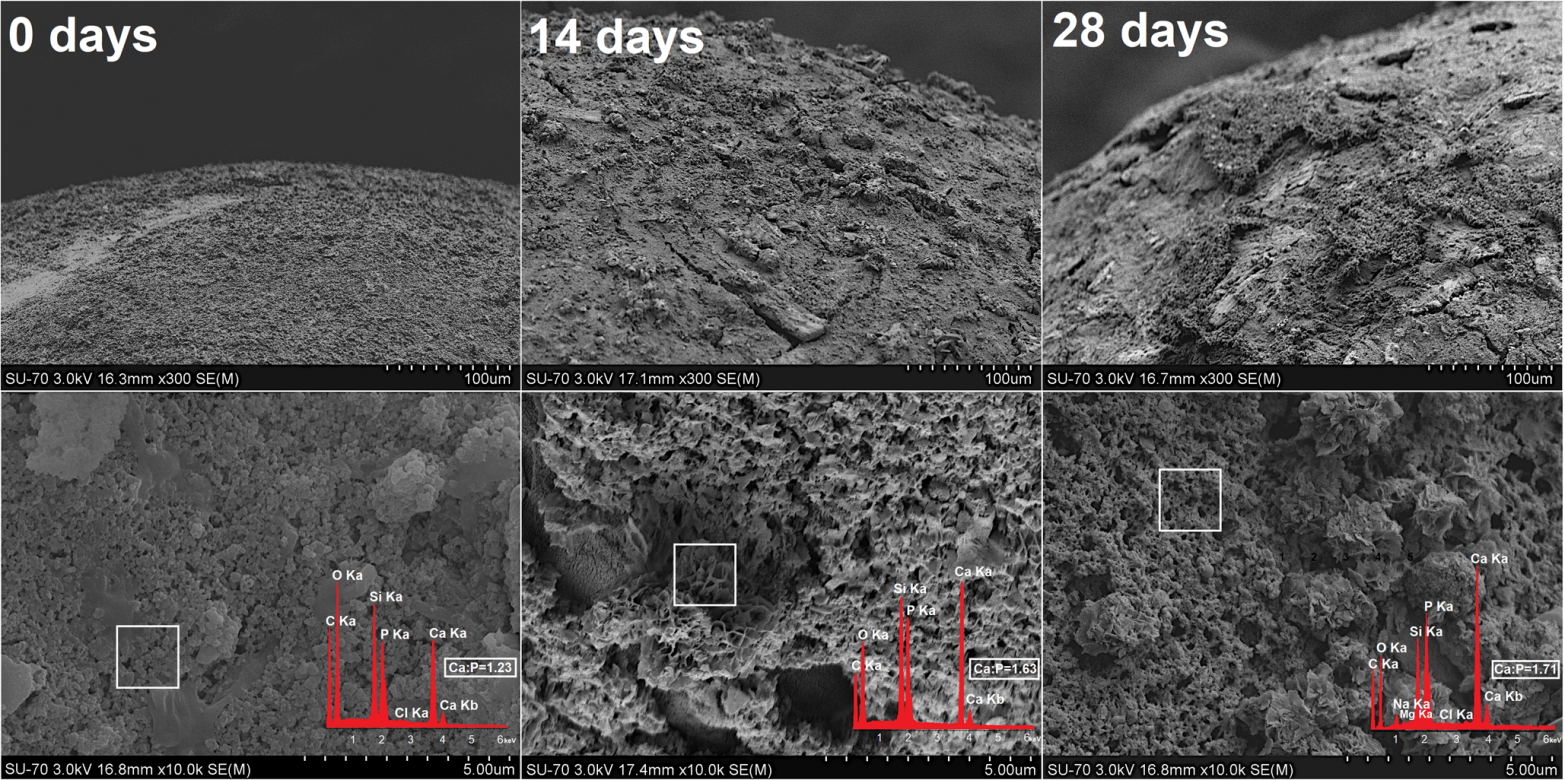
SEM-EDX micrographs of DOX-30CaPMSi pellets before and after 14, 28 days of mineralization assay in SBF

The morphological changes of DOX-30CaP@MSi pellets surface before and after 14 and 28 days of incubation in SBF are presented in Fig. 9. There was no significant difference between the pellet morphology before and after 7 days of mineralization assay (data not shown) what might be connected with both the drug release and the presence of excipients which impede the hydroxyapatite formation. After 14 days of mineralization assay, the new spherical particles with sizes in about 10–20 μm in diameter were formed on the pellets surface. After further 14 days of pellets incubation in SBF (28 days in total), the spherical particles formed closely to each other and reorganized into the continuous layer. Based on both the results obtained for powders and our previous reports [19, 20], the randomly formed clusters which spread over the pellets surface are characteristic for carbonate hydroxyapatite. Furthermore, the hydroxyapatite clusters changed their morphology after 28 days of immersion in SBF into porous, spongy-like one. Such morphology is characteristic for human cancellous bone [52]. The progressive formation of carbonate hydroxyapatite was also confirmed by EDX (Fig. 9) where the increased Ca:P molar ratio had been observed every 14 days of mineralization potential studies (from 1.23 to 1.71). The 1.71 Ca:P molar ratio and the presence of sodium, magnesium, carbonate and chloride ions in hydroxyapatite structure after 28 days of mineralization assay suggest that such composition is similar to composition of biological bone apatite [30].

## Conclusion

The combined effect of the mineralization potential of CaP minerals together with the high capacity drug loading of MSi for drug delivery is an outstanding perspective for bone therapy purposes. The rod-like-shaped CaP and spherical-shaped SiO_2_ structures successfully formed biphasic CaP@MSi composites structure with the low crystalline CaP phase by using wet precipitation method. The 30%CaP addition during the synthesis resulted in composites with the highest both mineralization potential *in vitro*, osteoblast biocompatibility, and successful drug loading.

The obtained pellets as proposed final form of the doxycycline-loaded composites allowed to complete 5-day release of drug with two-stage release profile which imitates the initial and maintenance doses of released drug. Such pellets may represent a novel solution in development of prolonged release bone drug delivery systems supporting the pharmacological treatment of *osteomyelitis*. After implantation, such system may provide the biphasic drug release in order to lower the doses of parenterally administrated antibiotics, thus reducing both the possible adverse effects and risk of antibiotic resistance. Additionally, obtained system seems to support the bone regeneration *via* surface hydroxyapatite formation. Extension of the presented experiments to antimicrobial activity of obtained formulations is in progress and will be reported in due course.

## Electronic supplementary material

Supplementary material 1 FTIR spectra of synthesised materials: 10CaP@MSi, 20CaP@MSi, 30CaP@MSi before (black) and after (red) the calcination with CTAB and hydroxyapatite reference samples. (PNG 555 kb)

High resolution image (TIF 640 kb)

Supplementary material 2 TEM micrographs of 100MSi, 10CaP@MSi, 30CaP@MSi and 100CaP materials. (PNG 375 kb)

High resolution image (TIF 23216 kb)

Supplementary material 3 N_2_ adsorption-desorption isotherms (a) and the pore-size distribution (b) with the summarized data of BET specific surface area, the total pore volume and average pore diameter of synthesised materials: 100MSi, 10CaP@MSi, 20CaP@MSi, 30CaP@MSi and 100CaP. (PNG 149 kb)

High resolution image (TIF 698 kb)

Supplementary material 4 (DOCX 13 kb)

Supplementary material 5 FTIR spectra of 30CaP@MSi material before and after (DOX-30CaP@MSi) DOX adsorption with DOX reference sample. (PNG 81 kb)

High resolution image (TIF 267 kb)

Supplementary material 6 SEM micrograph of DOX-30CaP@MSi pellet after the drug release studies. (PNG 96 kb)

High resolution image (TIF 3372 kb)
