# Supplementary figures and images for: Biphasic composite of calcium phosphate-based mesoporous silica as a novel bone drug delivery system
Source: Drug Deliv Transl Res. 2019 Dec 9;10(2):455–70. doi: 10.1007/s13346-019-00686-3 (PMC7066108; doi:10.1007/s13346-019-00686-3)

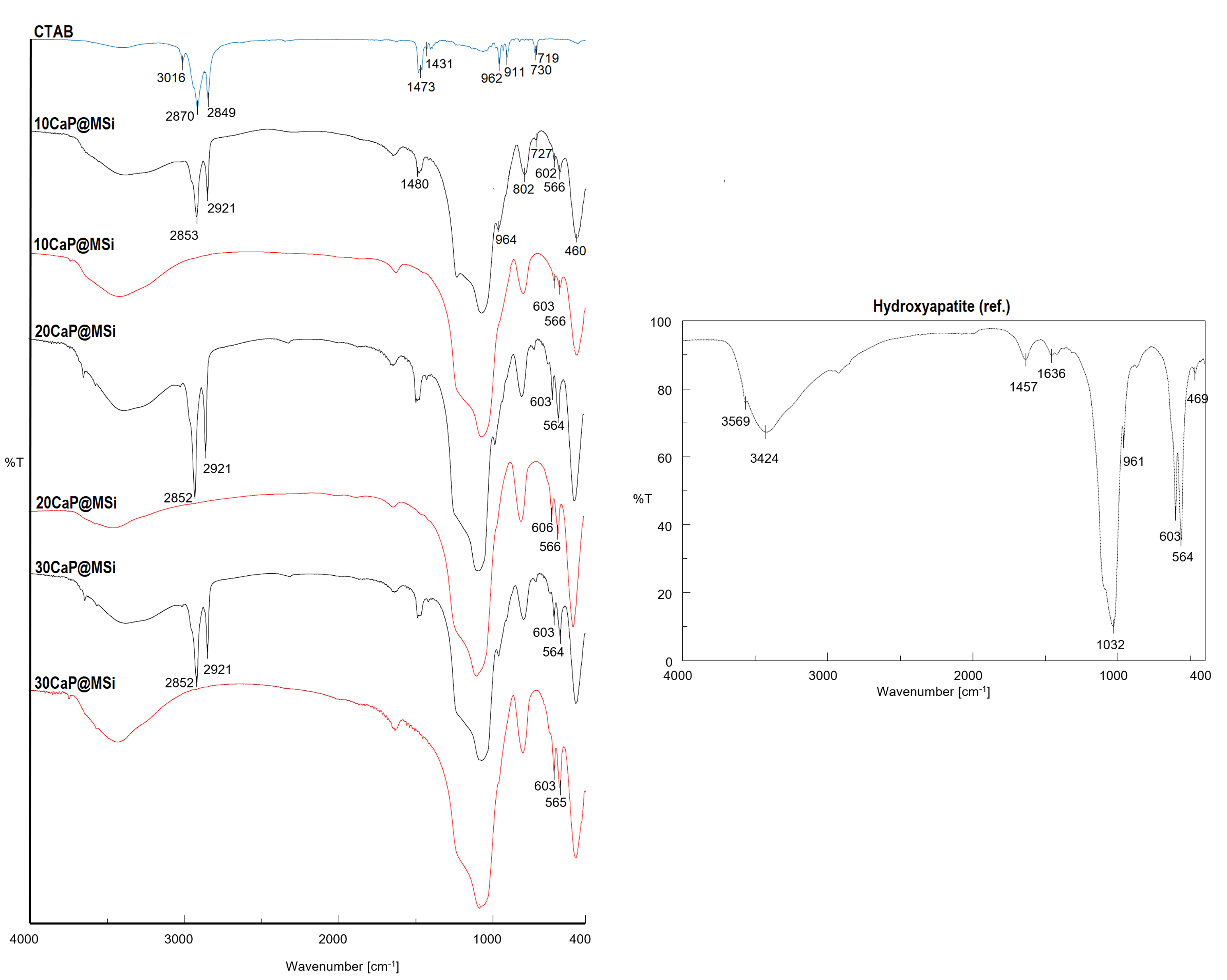

Supplement: Supplementary file 1 — FTIR spectra of synthesised materials: 10CaP@MSi, 20CaP@MSi, 30CaP@MSi before (black) and after (red) the calcination with CTAB and hydroxyapatite reference samples. (PNG 555 kb) [file 13346_2019_686_Fig10_ESM.png]

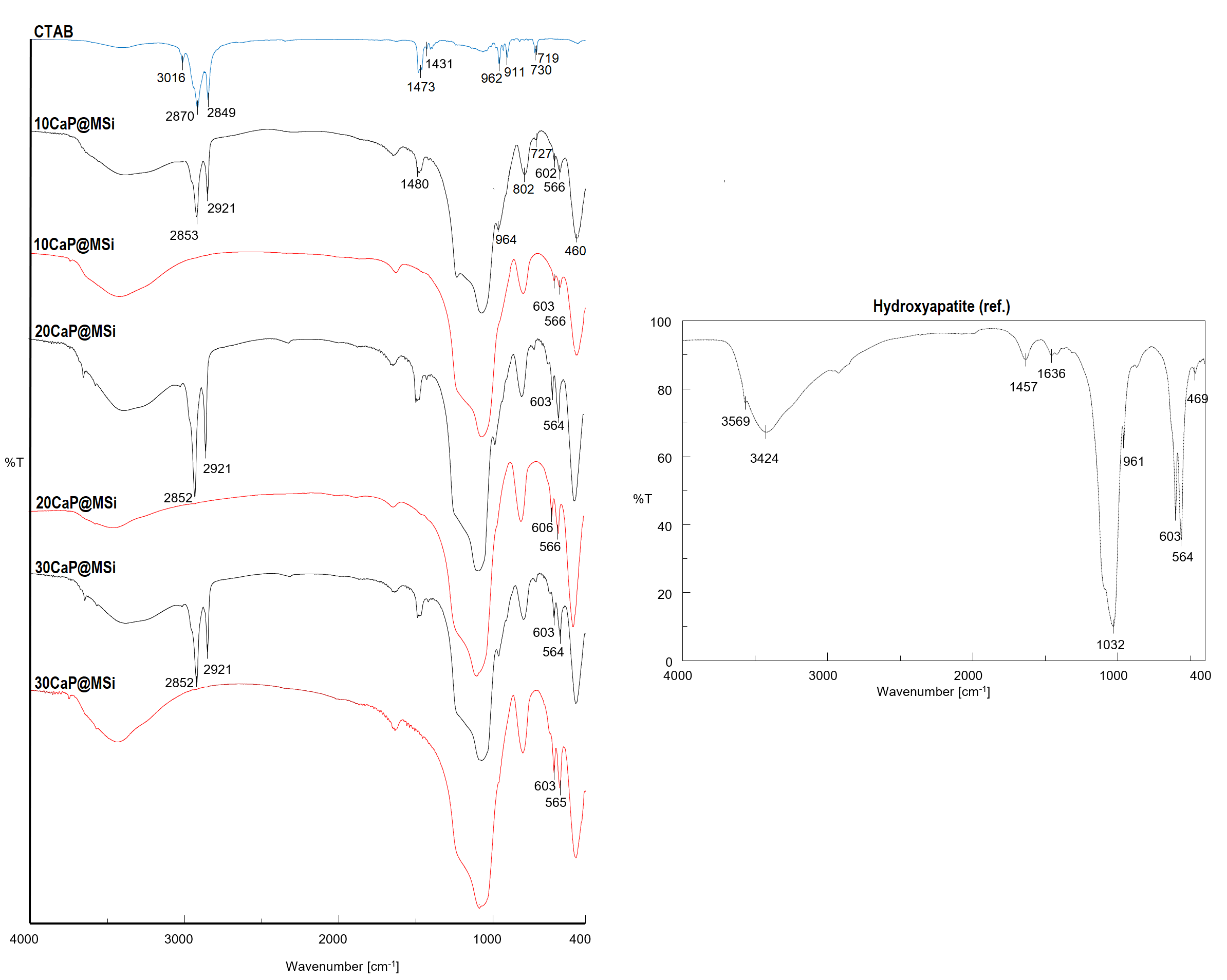

Supplement: Supplementary file 2 — High resolution image (TIF 640 kb) [file 13346_2019_686_MOESM1_ESM.tif]

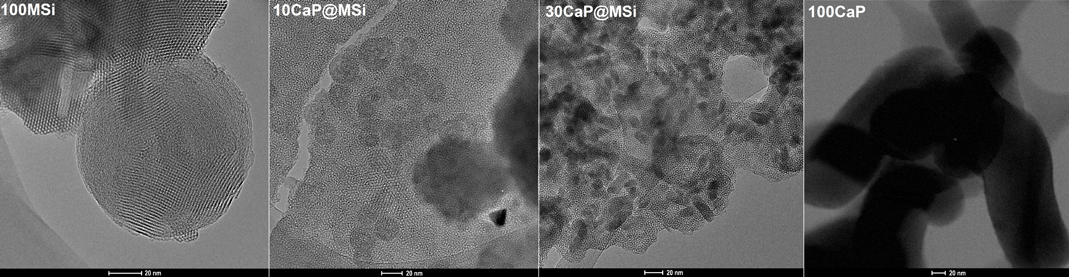

Supplement: Supplementary file 3 — TEM micrographs of 100MSi, 10CaP@MSi, 30CaP@MSi and 100CaP materials. (PNG 375 kb) [file 13346_2019_686_Fig11_ESM.png]

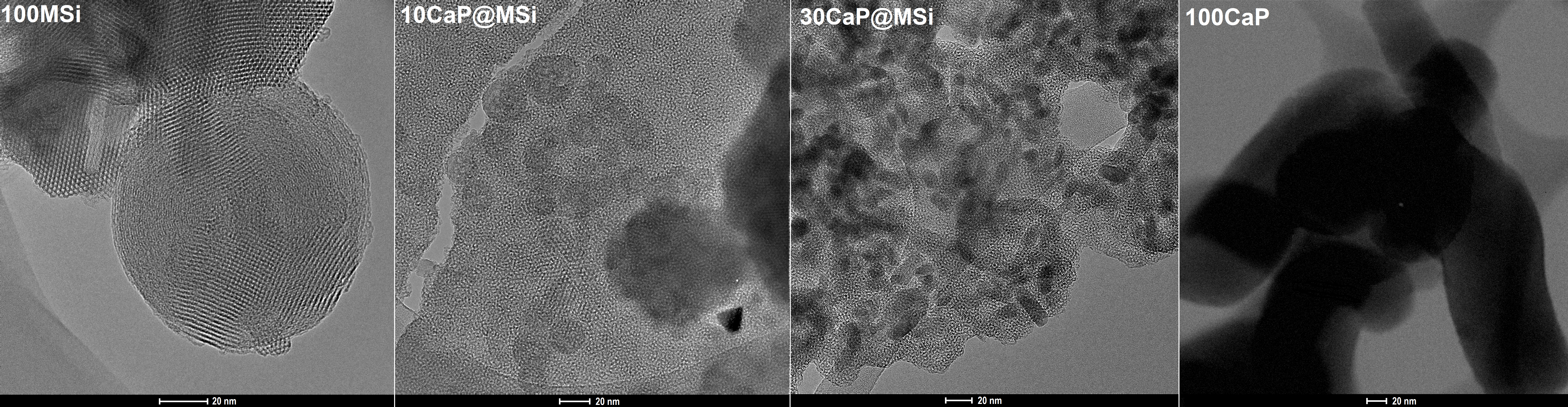

Supplement: Supplementary file 4 — High resolution image (TIF 23216 kb) [file 13346_2019_686_MOESM2_ESM.tif]

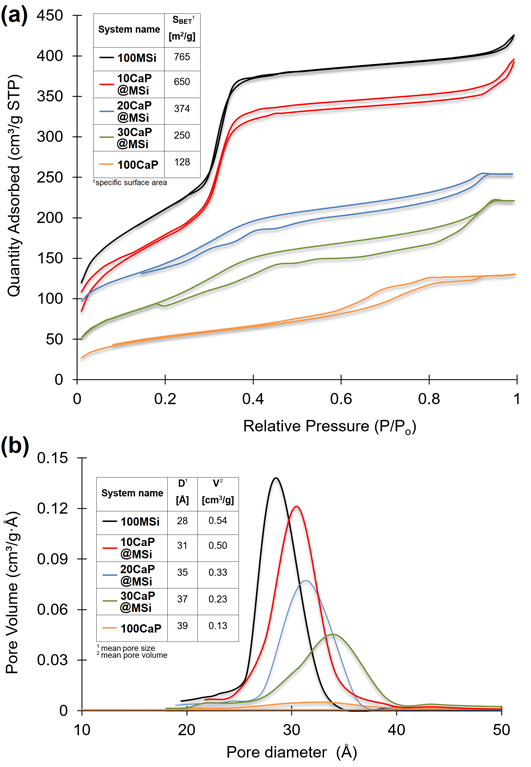

Supplement: Supplementary file 5 — N2 adsorption-desorption isotherms (a) and the pore-size distribution (b) with the summarized data of BET specific surface area, the total pore volume and average pore diameter of synthesised materials: 100MSi, 10CaP@MSi, 20CaP@MSi, 30CaP@MSi and 100CaP. (PNG 149 kb) [file 13346_2019_686_Fig12_ESM.png]

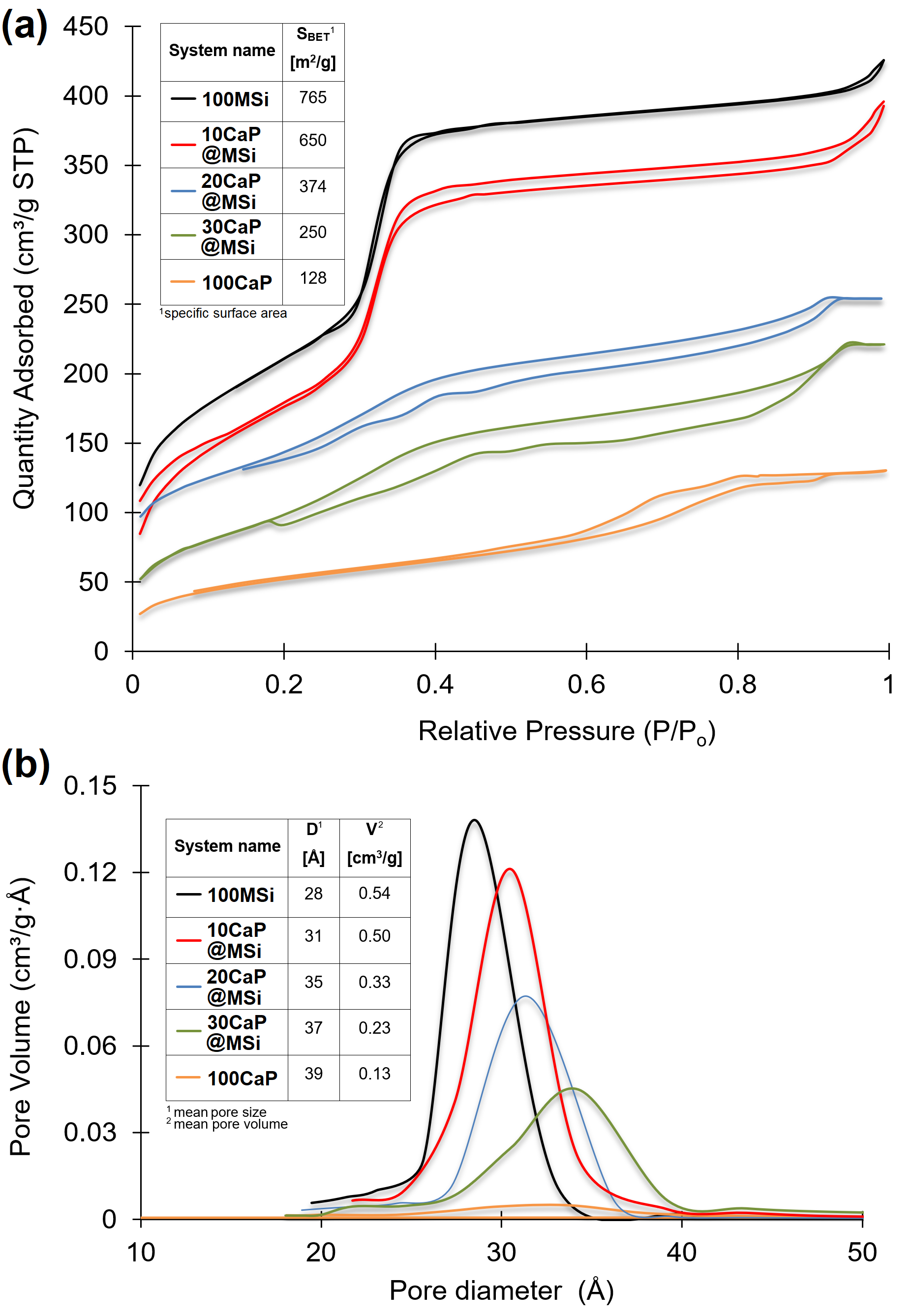

Supplement: Supplementary file 6 — High resolution image (TIF 698 kb) [file 13346_2019_686_MOESM3_ESM.tif]

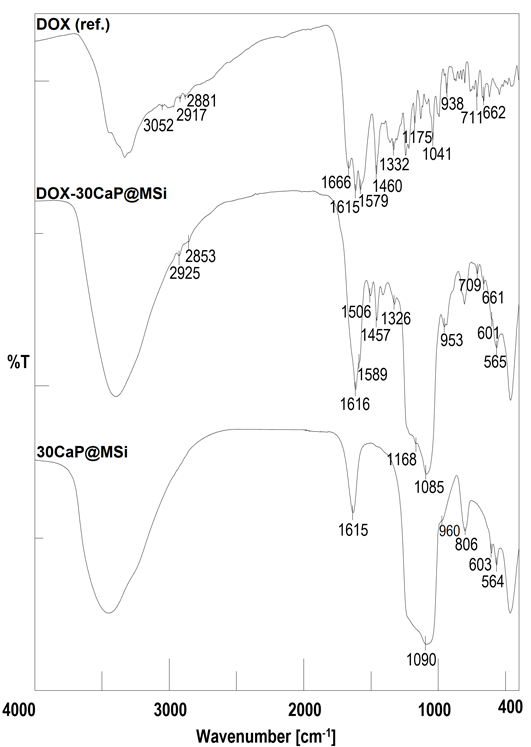

Supplement: Supplementary file 8 — FTIR spectra of 30CaP@MSi material before and after (DOX-30CaP@MSi) DOX adsorption with DOX reference sample. (PNG 81 kb) [file 13346_2019_686_Fig13_ESM.png]

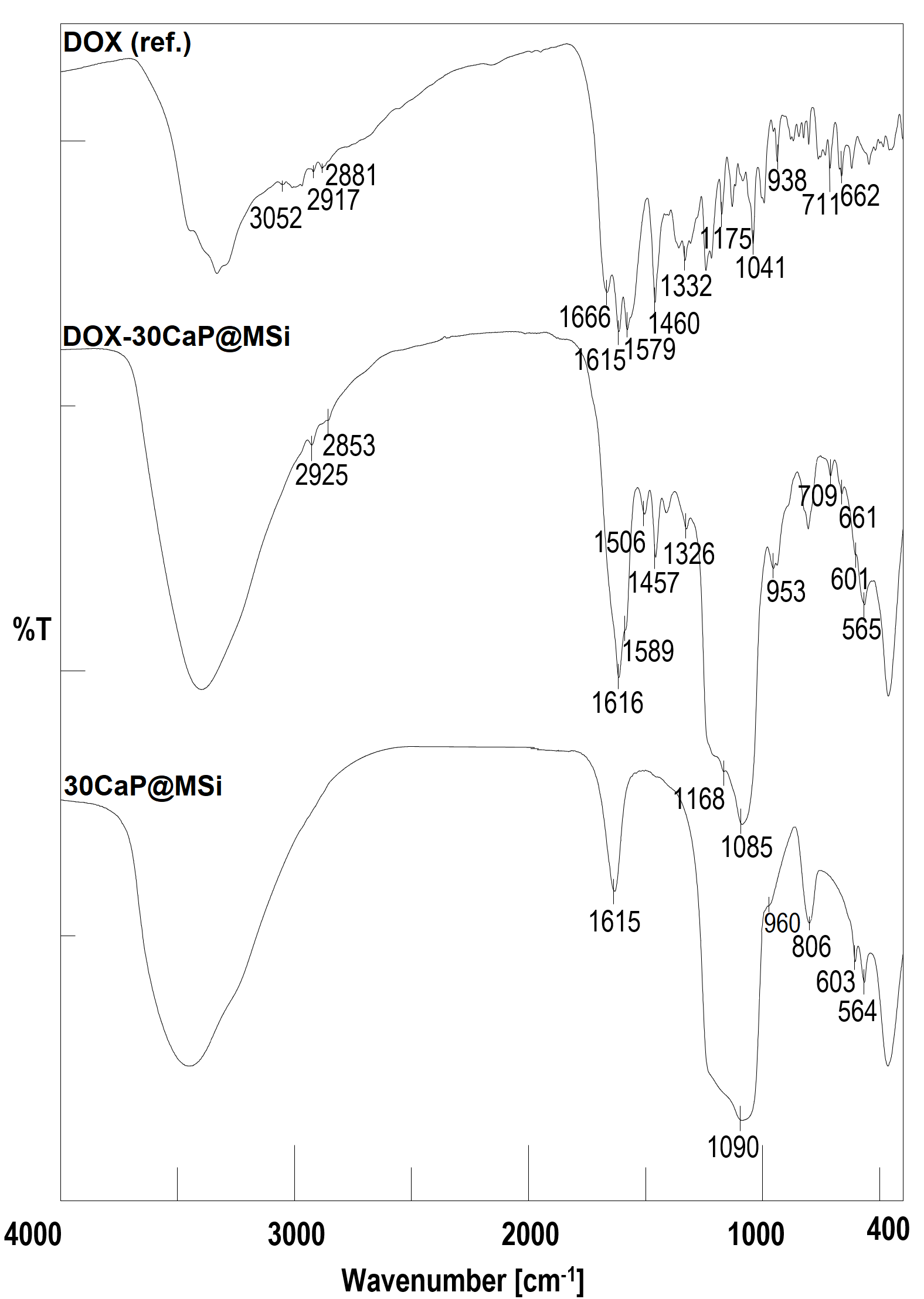

Supplement: Supplementary file 9 — High resolution image (TIF 267 kb) [file 13346_2019_686_MOESM5_ESM.tif]

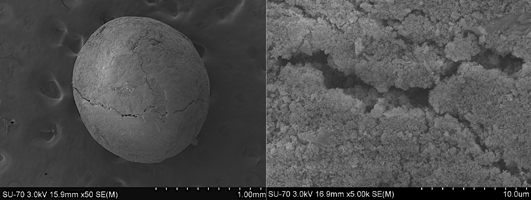

Supplement: Supplementary file 10 — SEM micrograph of DOX-30CaP@MSi pellet after the drug release studies. (PNG 96 kb) [file 13346_2019_686_Fig14_ESM.png]

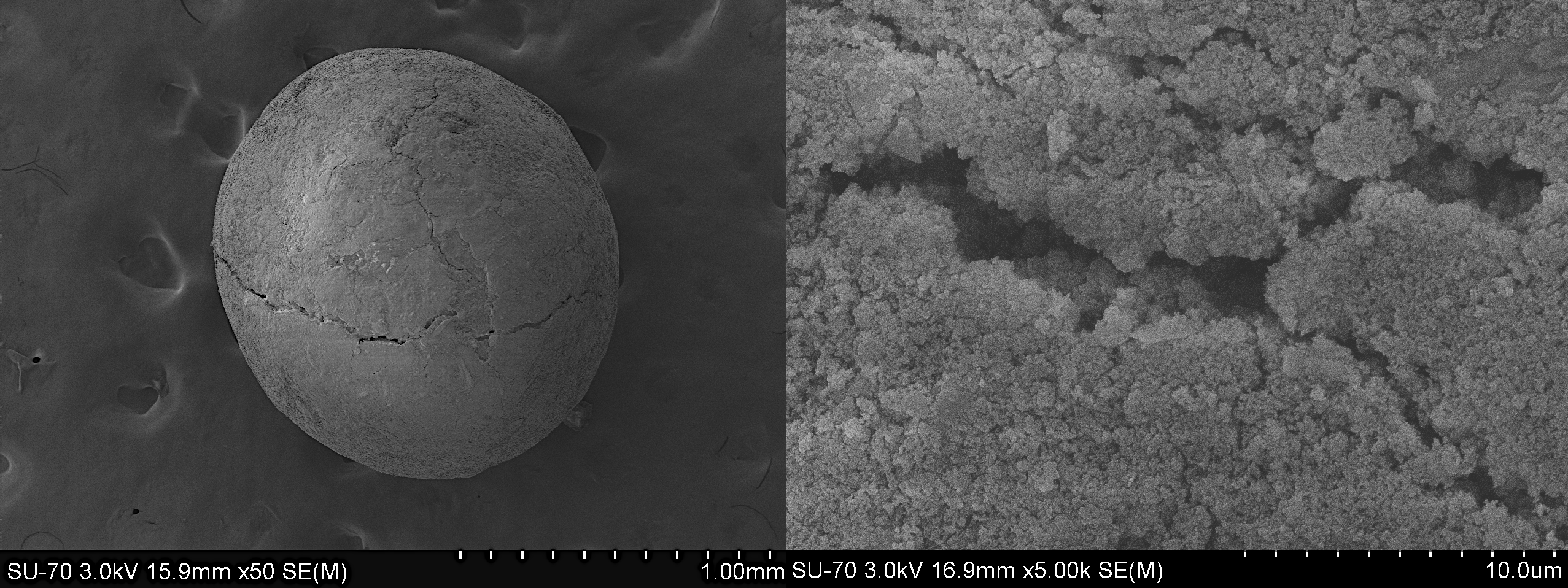

Supplement: Supplementary file 11 — High resolution image (TIF 3372 kb) [file 13346_2019_686_MOESM6_ESM.tif]
