# Supplementary material for: Biphasic composite of calcium phosphate-based mesoporous silica as a novel bone drug delivery system
Source: Drug Deliv Transl Res. 2019 Dec 9;10(2):455–70. doi: 10.1007/s13346-019-00686-3 (PMC7066108; doi:10.1007/s13346-019-00686-3)
Supplement: Supplementary file 7 — (DOCX 13 kb) [file 13346_2019_686_MOESM4_ESM.docx]

**Supplementary material 4**

Molar ratios of Ca/P for CaP@MSi as a function of the time of immersion in SBF solution

| **Time**  **[days]** | **10CaP@MSi** | **20CaP@MSi** | **30CaP@MSi** | **Commercial Hap** |
| --- | --- | --- | --- | --- |
| **0** | 1.05 | 1.12 | 1.23 | 1.67 |
| **7** | 1.35 | 1.48 | 1.67 | 1.67 |
| **14** | 1.43 | 1.50 | 1.67 | 1.67 |
| **21** | 1.53 | 1.62 | 1.67 | 1.67 |
| **28** | 1.60 | 1.65 | 1.70 | 1.67 |
